# HIV in (and out of) the clinic: Biomedicine, traditional medicine and spiritual healing in Harare

**DOI:** 10.1080/17290376.2014.938102

**Published:** 2014-05-12

**Authors:** Stephen O'Brien, Alex Broom

**Affiliations:** ^a^ PhD Sociology, is a tutor at the School of Humanities and Social Science, The University of Newcastle, Newcastle, Australia; ^b^ PhD, is Associate Professor of Sociology, ARC Future Fellow, School of Social Science, The University of Queensland, St Lucia, Brisbane, Australia

**Keywords:** antiretroviral therapy, HIV/AIDS, qualitative sociology, spiritual healing, traditional medicine, Zimbabwe, thérapie antirétroviale, guérisseurs par la foi, guérison traditionnelle, sociologie qualitative, VIH/SIDA, Zimbabwe

## Abstract

Contemporary lived experiences of the human immunodeficiency virus (HIV) are shaped by clinical and cultural encounters with illness. In sub-Saharan countries such as Zimbabwe, HIV is treated in very different ways in various therapeutic contexts including by biomedical experts, traditional medicine and faith healers. The co-existence of such expertise raises important questions around the potencies and limits of medicalisation and alternative healing practices in promoting HIV recovery. First, in this study, drawing on in-depth qualitative interviews with 60 people from poor urban areas in Harare, we explore the experiences of people living with and affected by HIV. Specifically, we sought to document, interrogate and reflect on their perceptions and experiences of biomedicine in relation to traditional medicine and spiritual healing. Their accounts indicate that traditional medicine and spiritual beliefs continue to significantly influence the way in which HIV is understood, and the forms of help and care people seek. Second, we observe the dramatic and overwhelmingly beneficial impact of Antiretroviral Therapy and conclude through Zimbabwean's own stories that limitations around delivery and wider structural inequalities impede its potential. Lastly, we explore some practical implications of the biomedical clinic (and alternative healing practices) being understood as sites of ideological and expert contestation. This paper aimed to add to our knowledge of the relationships between traditional medicine and spiritual healing in connection with biomedicine and how this may influence HIV treatment and prevention.

## Résumé

Les experiences contemporaines du virus humain de deficience immunologique VIH sont determinees par des rencontres cliniques et culturelles avec la maladie. Dans les pays sub-Sahariens comme le Zimbabwe, le VIH est soigné de manière très différente selon des contextes thérapeutiques variés y compris les experts en médecine biomédicale, les guérisseurs traditionnels et les guérisseurs par la foi. L'existence côte à côte de ces pratiques soulève des questions importantes concernant l'efficacité et les limites de la médicalisation et des pratiques de guérison alternatives afin de promouvoir la guérison du VIH. Dans cette étude faisant appel à des interviews qualitatives en profondeur avec 60 personnes provenant de secteurs urbains appauvris à Harare, nous explorons les expériences des personnes qui vivent avec et qui sont affectées par le VIH. Surtout nous avons essayé de documenter d'interroger et de reflechir sur leurs perceptions et expériences de la biomédicine par rapport a la medicine traditionnelle et des guérisseurs par la foi. Leurs comptes rendus indiquent que les pratiques traditionnelles et les croyances spirituelles continuent à influencer de façon significative la façon dans laquelle le VIH est compris et les types d'aide et de soins que les gens recherchent. Deuxiemement, nous observons l'impacte drammatique et bénéfique de la Thérapie Antirétroviale (TAR) et concluons selons les rapports du Zimbabwe que les limites de livraison et que des inégalités de structure répandues empêchent l'accomplissement de leur potentiel. En dernier lieu, nous explorons certaines implications pratiques de la Clinique biomédicale (et autres pratiques ‘alternatives’ de guérison) comme etant comprises en tant que champs idéologiques et experts en contestation. Le but de cet article est de renforcer notre connaissance de la relation entre la guérison traditionnelle et la guérison par la foi en rapport avec la biomédicine et comment cela peut influencer le traitement et la prévention du VIH.

## Introduction

In developing countries such as Zimbabwe, the human immunodeficiency virus (HIV)^1^ may be treated by practitioners who come from traditions as varied as biomedicine, traditional medicine and faith healing. Such a co-existence raises important questions concerning the potencies and limitations of various healing practices in promoting HIV recovery. While the advent of antiretroviral therapy (ART) has impacted favourably on stigma (Campbell, Skovdal, Madanhire, Mugurungi, Gregson & Nyamukapa 2011) as well as patient health (Campbell, Scott, Madanhire, Nyamukapa & Gregson 2011), its relatively late arrival in Zimbabwe meant that ART was introduced into a context where traditional therapeutic systems were pre-existing and well established. While the significance of traditional/spiritual healing and HIV has been well demonstrated in the Zimbabwean context (Gregson, Zhuwau, Anderson & Chandiwana 1999; Rödlach 2006; Simmons 2002; Taylor 2005), there has been less research on the life experience of patients who combine the various HIV therapies. To this end, this paper focuses on the accounts of 60 people living in the economically poor areas of Harare, the capital of Zimbabwe, to explore the participants' perceptions and experiences of biomedicine in relation to traditional medicine and spiritual healing. Specifically, we interrogate how cultural influences shape understandings about HIV and the forms of help and care that people seek. While the overwhelmingly beneficial impact of ART is clearly evident in the dramatic recoveries that are related by the participants, their accounts are tempered by concerns about logistical, clinical and administrative problems associated with ART. These limitations also figure in a discussion around how the biomedical clinics (and alternative healing practices) can be understood as sites of ideological and expert contestation. This paper aims to add to our conceptualisation of the relationships between traditional medicine and spiritual healing in relation to biomedicine and concludes by considering what this may imply for clinical practices, community education and public health.

## Background

### A brief note on the history and emergence of HIV in Zimbabwe

The national health system that Zimbabwe inherited at independence in 1980 had been primarily structured around delivering acute, curative services for a small urban white minority (Sanders & Davies 1988). Despite efforts to expand and reorient attention towards primary health care in order to serve a broader section of the population (Loewenson, Sanders & Davies 1991), the new Government was unprepared for an epidemic which was to cause two million acquired immune deficiency syndrome (AIDS) deaths and an additional one million or more HIV infections (Ministry of Health and Child Welfare [MoHCW] 2009). From the beginning of the outbreak, the official response to HIV was largely uncoordinated and constructed around denialism (i.e. not a serious public health problem) and blame (i.e. only the sexually deviant are affected) (Duffy 2005). In addition, Government health policy forbade ‘AIDS’ to be written ‘as a cause of death’ (Ministry of Health [MoH] 1987:22), and the number of officially recognised cases was minimised and kept secret (Rödlach 2006). Ministry guidelines defined those at risk as ‘patients with multiple sexual partners … promiscuous persons and prostitutes’ (MoH 1987:8), which reinforced an emerging sexual deviance paradigm in relation to HIV transmission by implying that women were the ones responsible for HIV's spread (Bassett & Mhloyi 1991). When the disease was taken more seriously, the resources to implement an effective biomedical response were inadequate as from the mid-1980s health funding progressively declined and people stopped using health services (Poverty Reduction Forum [PRF] 2004). Given this situation the prognosis for those Zimbabweans who knew their HIV status was poor and their treatment options limited to herbal remedies, prayers, exorcism rites and the lifestyle and dietary proscriptions of positive living. For most people who were HIV positive, their eventual progression to AIDS and death within 10 years would remain inevitable until after 2004 when public health authorities started to prescribe ART.

### ‘New’ biomedical therapies

While effective in halting disease progression and promoting dramatic patient recoveries, ART carried some physical, emotional and economic cost for the recipients (Nixon, Hanass-Hancock, Whiteside & Barnett 2011). Patients were subject to a long-term and techno-scientific model of disease control that involved consuming specific combinations of potentially toxic synthetic drugs in accordance with a strict adherence regime (Skovdal, Campbell, Madanhire, Mupambireyi, Nyamukapa & Gregson 2011). As ART reduces rather than eliminates HIV, the body (and thus the person) is required to coexist with the virus on a long-term basis, transforming a fatal and acute illness into a long-term chronic condition (Nixon *et al*. 2011). To be most effective, ART requires the collection of a range of empirical body function data. These include kidney and liver tests; viral load (the amount of virus in the blood) as well as chest x-rays and pregnancy tests and monitoring for potential co-morbidities such as tuberculosis (TB), meningitis, hepatitis, sexually transmitted infections and HIV levels (MoHCW 2010). In addition, before enrolment in ART the patients are assessed to gauge their attitudes to counselling, disclosure and drug adherence (MoHCW 2010). The biomedical clinic, in theory, therefore offers an integrated care package involving a suite of monitoring technologies operated by a full complement of specially trained doctors, nurses, counsellors, technicians and rehabilitation specialists. However, as the responses of the participants in this study illustrate, this ideal is more aspirational than reality. With the exception of a few individuals who had private or privileged access to drugs, and a small cohort involved in clinical trials, the first Zimbabweans to receive ART were an initial group of 15,000 who were enrolled in late 2004 (Apollo, Takarinda, Mugurungi, Chakanyuka, Simbini & Harris 2010). Potential recipients were identified from hospitals, testing centres and health programmes such as Ante Natal Care, TB and sexual health clinics, the main criteria for enrolment being a CD4 cell count under 200 (MoHCW 2010). A CD4 level between 500 and 1200 is normal but in the absence of ART, as these cells are destroyed by the virus, the immune system is seriously weakened and the body left vulnerable to infection (MoHCW 2010).

Since 2004, the number of people enrolled on ART has steadily increased to reach 150,000 in 2008, and 280,000 people in 2010, but still only reaches about half of those in need (Apollo *et al*. 2010; Government of Zimbabwe [GoZ] 2012). This reflected the new guidelines announced in 2010 which revised the CD4 entry level to 350 (Apollo *et al*. 2010) and which meant that the overall number of those needing ART also increased to around 600,000 (MoHCW 2010). The overall emphasis of the treatment effort has been to provide medication to the maximum number of people in preference to ongoing follow-up. As a consequence ART is now delivered in Zimbabwe, as in most Africa countries, with significantly less technologisation (i.e. health personnel, laboratories and medical facilities, etc.) than occurs in Western contexts. Conceptual and practical issues relating to the mass roll out of ART include the dilemma of routine versus needs-based patient monitoring (DART Trial Team 2010; Hallett, Gregson, Dube, Mapfeka, Mugurungi & Garnett 2011), the orientation of patient care towards the family and community (Colvin 2011) and the underlying social and economic contexts of treatment and prevention (Nguyen, Bajos, Dubois-Arber, O'Malley & Pirkle 2011). Zimbabwe-specific ART-related research deals with issues such as drug resistance (Tshabalala, Manasa, Zijenah, Rusakaniko, Kadzirange, Mucheche, *et al.* 2011), social obstacles to enrolment and adherence (Mhlanga-Gunda 2010; Skovdal, Campbell, Nhongo, Nyamukapa & Gregson 2011; Skovdal, Campbell, Nyamukapa & Gregson 2011), staff/patient interactions (Campbell, Scott, *et al*. 2011) and stigma (Campbell, Skovdal, *et al*. 2011). Less research has focused on the lived experience of intersections between the new health technologies and the often shifting terrain of religious belief and its influence on traditional medicine and metaphysical notions of illness and healing, themes which are explored in this paper.

### ‘Old’ beliefs

A variety of religious and spiritual philosophies with different conceptions of healing coexist in Zimbabwe. Broadly speaking they can be grouped into four categories: traditional religious beliefs and practices that were carried over from the pre-colonial era, mission churches (Catholic and Protestant), African Christian or spirit churches (also known as Apostolics or Vapostori) and Pentecostal ministries. While other faiths and non-believers are present in Zimbabwe (Zimbabwe National Statistics Agency [ZIMSTAT] & ICF International 2012), almost all of the participants identified with one or more of these main belief systems. The Shona peoples who form the main ethnic group in Harare, and in north and north-west Zimbabwe, historically ascribed to a cosmology constructed around the power which is held by ancestral spirits over health and well-being (Bourdillon 1997). For example, if the inhabitants of the spirit world, to which the living pass after death, are displeased (e.g. through failure by mortals to follow customs), the ancestral spirits may seek revenge on the living (Bourdillon 1997). Serious and prolonged illness with no obvious cause, or ongoing tension and stress in the community, may therefore lead the afflicted to consult a *n'anga* (traditional medical practitioner) in order to establish communication with the spirit world, identify and remedy the problem (e.g. through traditional medicines, rituals and witchcraft exposure) (Shoko 2011). Shona healing and healers do not necessarily reject biomedicine but regard their own methods as the best way to deal with the underlying cause of serious illness (Gregson *et al*. 1999). Evolving out of small-scale agriculture and village life, Shona tradition has adapted to capitalism and urbanisation and many of its customs have survived and continue to influence people's overall outlook on life (Bourdillon 1997). Bride price or *lobola*, for example, now involves monetary payments (where once it involved the exchange of implements or cattle) and *n'angas* practice in the cities in return for cash (Duffy 2005; Simmons 2002:109).

Christianity was introduced into Zimbabwe in the early colonial period by missionaries who established schools, churches and hospitals and zones of denominational influence (Ndlovu-Gatsheni 2009). The mainstream or mission churches still operate over 100 clinics and hospitals and play a role in providing rural and HIV-related health services (PRF 2004). In comparison to these churches the Pentecostals, with their modernistic individualist-oriented theology, are a more recent and expanding phenomenon (Maxwell 2000). They preach against the ‘old ways’ in favour of healing through faith, prayer and exorcism (Maxwell 2000). The Apostolic or Vapostori Churches are also opposed to some aspects of traditional culture but endorse others such as polygamy, prophecy and the spiritualisation of illness (Scarnecchia 1997). Vapostori adherents are known for their open air worship, their distinctive white garb and, for some orthodox adherents, a rejection of biomedical care and treatment (Bourdillon 1997). Regardless of the different forms that religious beliefs take, they all influence and mould cultural perceptions of HIV on various levels both as an illness (i.e. how the sick are treated therapeutically) and as a personal and behavioural outcome (i.e. morality, sexuality and gender). While the main faith systems have different points of emphasis, their various community rituals and practices (taboos, discriminations, proscriptions, etc.) shape the ways in which lives are experienced and choices made (e.g. forms of treatment and attitudes to healing).

## Methods

Community-based AIDS Service Organisations (ASOs) were utilised to recruit participants from the four areas of Harare in which they each operated: Mbare (old inner centre), Glenview (industrial residential), Epworth (peri-urban informal) and Chitungwiza (satellite dormitory). Greater Harare has a population of 2.5 million (ZIMSTAT 2013). Each location, while distinct, was characterised by poverty, intermittent utility services, water borne diseases and large populations (ZIMSTAT 2013). The selection of individuals involved a combination of purposive and theoretical sampling strategies as participants were nominated by the leaders of each ASO on the basis of having experiences relevant to the research question (Bryman 2004). Relevance in this context meant living in an urban area, being HIV affected or infected and involved with or linked to an ASO. Apart from suggesting to the leaders of each ASO that they nominate 15 people from each of their organisations/areas who reflected different genders, religious affiliations and ages, the researchers did not become directly involved in the recruitment process.

On this basis, the participants were recruited by word of mouth, text messages and cell phone calls. A target of 60 participants was set as we believed that this number would provide a broad representation of each township and allow us to achieve data saturation across the overall sample and help to minimise bias (Glaser & Strauss 1973). The final cohort was composed of 25 men and 35 women. This ratio was perhaps indicative of the greater willingness of women to be involved in HIV-related programmes. This trend was mentioned incidentally by the majority of participants during the interviews. The commonality between the ASOs was their involvement in the Zimbabwe Social Forum.

Participants were not directly asked their HIV status in order to respect the voluntary nature and confidentiality of disclosure in both the recruitment process and the interviews. Furthermore, during the analysis each person was referred to by a unique pseudonym, mainly drawn from the Anglicised names which many Shona people have. While participants were not remunerated for their time, they were reimbursed for their transport costs. Follow-up interviews were conducted with several participants in 2011 in order to clarify specific points made in the original meeting. The average age of participants was 39 years, with the youngest being 18 and the oldest 69 years. Most were in the 31–45 age group and 46 of the total revealed that they were HIV positive, with just over half either enrolled on ART or in the process of enrolment. While participants were not chosen on the basis of their ethnicity, all spoke Shona and understood English. Eight reported that they had at least one parent or grandparent who had migrated from the neighbouring countries and one person identified as Ndebele, the second main ethnic group mainly based in the South West of the country.

We utilised a qualitative design primarily, based on semi-structured interviews given the need to explore in depth people's lived experiences. This method proved to be appropriate as some participants were unwilling to disclose their status and hence disinclined to participate in focus group discussions. The interviews were structured around the following broad themes: views and perspectives of HIV and the community context in which they were held; individual experiences of stigma and discrimination; access to treatment and understandings about transmission. In order to minimise apprehension and anxiety regarding dialogue about HIV status, the interviews were conducted in a trusted space, the offices of a legal aid non-government organisation (NGO). Interpreting was available and respondents were given the option of speaking in Shona. The interviews, which lasted from 45 to 90 minutes, were conducted by the author and the research assistant (a Shona-speaking Zimbabwean). Consent forms, which assured confidentiality and the right to withdraw from the research at any point, were provided in both English and Shona. They were read aloud to each participant before he or she was asked to sign.

As we adopted a reflexive approach to the data gathering (Ezzy 2002), the interview schedule and questions were reviewed on the basis of emerging evidence and tailored to the situation of each person. Field notes were compiled and the discussions were accordingly recorded and transcribed in full by the principal researcher and the research assistant. Ethics clearance was obtained from the authors' university, the National Research Council of Zimbabwe, and letters of support were provided by the four ASOs. During the fieldwork the primary author systematically appraised the data, identified key themes and applied a series of codes to develop an overall generalised framework for data analysis within the interpretative traditions of qualitative research (Charmaz 1990; Ezzy 2002).

## Results

The inductive thematic approach to data gathering and analysis resulted in the emergence of various themes: stigma, culture, gender and social mobilisation, as well as how the experience of HIV is mediated by biomedicine and metaphysical notions of healing. While recognising that HIV affects people irrespective of their income, geography, age, gender, etc., this study focused on marginalised populations as we considered that the poor in Zimbabwe face the greatest challenges and obstacles to accessing health care. This is especially so given the deterioration in recent years of social indicators such as household poverty, child malnutrition and unemployment (Apollo *et al*. 2010; GoZ 2012).

### The cultural construction of HIV

The persistence of erroneous ideas about HIV transmission in Zimbabwe is illustrated by the Demographic Health Surveys (DHS) which have been conducted five times since the 1980s and most recently in 2010−2011 (ZIMSTAT & ICF International 2012). According to these surveys, Zimbabweans have near-universal awareness of HIV and AIDS; however, as the participants' comments also revealed, there are significant limitations to this knowledge. For example, some participants expressed ambivalence about the role of condoms in HIV prevention: ‘they break when you are in action’ (Mark), ‘People claim that they are using them but you see their wives pregnant’ (Havison), ‘some are allergic to condoms’ (Havison), ‘they reduce the manhood’ (Helen), ‘the oil that is in the condoms is … helping spread the HIV’ (Bertha) and ‘they are expired or they are not electronically tested’ (Gwyn). These views provide some context to the 2010–2011 DHS finding that only about half of the respondents had comprehensive levels of knowledge about HIV transmission, especially the role of condoms in preventing infection. Moreover, 10% of DHS respondents indicated that they thought it possible to contract HIV from witchcraft or other supernatural means (ZIMSTAT & ICF International 2012). These misunderstandings are also present in our findings:

[churches] say that … sin causes a person to be infected … at one workshop I attended they didn't encourage people to use condoms … they said they didn't want to teach about condoms [as it] … would encourage promiscuity … . (Jacob, man aged 41, HIV positive)

[according to] some of the pastors … a spiritual force makes someone go and have sex with a person who has a fungal infection or these HIV infections. So it is a spirit that pushed some to do that evil…. (Sinai, man aged 39, HIV positive)

 … [my family holds] strong traditional beliefs so the relatives are saying that ‘You deserve that [HIV]’ … some people believe that being HIV positive is because of disobedience to your parents, and the churches believe that maybe these are evil spirits … . (Joy, woman aged 45, HIV positive)

 … if someone was sick they … would say it is witchcraft and they have got this story about … goblins that can suck your blood so you start getting thin… so they would … take the person to a traditional healer. (Maria, woman aged 42, HIV positive)

As can be observed in these quotes, the participants tended to refer to supernatural beliefs indirectly (‘the churches believe’, ‘the family says’, ‘the pastor teaches’ and so forth). Despite this reluctance to identify personally with notions of witches or other spiritual beings, it was clear that underlying supernatural ideas were present as the participants' accounts contained a mixture of competing cosmologies. According to Bourdillon (1997) and Ashforth and Nattrass (2005) respondents tend to under-report or downplay their familiarity with witchcraft and similar phenomena. In this context another useful insight from the 2010–2011 DHS was the fact that around half of the respondents thought that a positive person can be identified by the person's physical appearance. The long dormancy of HIV obscures the link between the events of infection and the onset of AIDS, and signifies that the experience of illness may be quite distinct to the diagnosis of disease and vice versa.

In contrast, supernatural understandings present a closer temporal association between illness and diagnosis. Jacob, for example, intimated that the virus is the result of Christian ‘sin’, whereas Sinai attributed the means of entry into the body to ‘possession by evil spirits’. In other words, these and other accounts suggest that the existence of HIV may be accepted but the process of infection is spiritualised. In Shona tradition this may be a result of the actions of malevolent witches and goblins or angry ancestral spirits (Bourdillon 1997). In recent years, witchcraft has become an indictment used mostly against women (Schmidt 1992) and this has fed into the idea that women spread HIV because of their alleged failure to submit to (male dominant) tradition and culture (Duffy 2005). In an overall sense women in Zimbabwe are largely socially subordinate with lower incomes, fewer employment opportunities and poor educational participation rates (GoZ 2010). These and other social vulnerabilities reflect the gendering of cultural representations of illness and reduce women's ability to refuse unsafe sex, thereby contributing to the reality that females are 50% more likely to be HIV positive than males (MoHCW 2009). An illustration of the cultural blame ascribed to women can be observed in the following:

 … people don't understand that you can be HIV positive even if you are not a prostitute because they say if you have got that disease maybe you have been changing partners which is not true … especially the relatives like when my husband passed away they thought that I was the one who brought the disease … . (Gwyn, woman aged 37, HIV positive)

Gwyn believed that her in-laws accused her of having ‘brought the disease’ into the relationship by supposedly having multiple partners. Underlying this attribution of responsibility is the more subtle accusation, as had been expressed to Gwyn by her late husband, that witchcraft was to blame for their misfortune. This form of belief implies that metaphysical agents are responsible for HIV infection and ultimately AIDS. If the origin of disease is not framed biomedically, then biomedical treatment is less likely to be appreciated or enacted. In our study over one half of the participants reported having sought help for various physical and emotional ailments from traditional medicine and spirit mediums, faith healers (Pentecostal and Vapostori) or sacred artefacts (e.g. Catholic holy water). All of these distinctly framed the way HIV was understood and treated:

 … some of these pastors they [are] … saying that if you give your faith in God you will pray and the virus will go…. (Sinai, man aged 39, HIV positive)

[the Vapostori prophets] say ‘You go and bring a two-litre bottle of water’… They would bless this water and they would give you a prescription … ‘bathe with this water’ or ‘drink this water after every meal’, or sometimes you will have some stones, they will bless those stones they will say ‘Put that stone in the bucket of water when you bathe’. (Helen, woman aged 43, HIV positive)

Metaphysical healing practices take different forms according to the respective belief systems. Spiritual healing, for example, is mainly performed by the Pentecostal and Apostolic Churches as demonstrated here:

 … many people are opting for the prophets, those guys who gather around in marshes and swamp areas and in the open … so normally I see people … taken there, sick people in wheelbarrows and in commuter omnibuses … . (Byron, man aged 25, HIV status not declared)

 … if you continue maybe disclosing that you are HIV positive they think that you are hard hearted or have got a hard brain. (Joy, woman aged 45, HIV positive)

 … even to my [Vapostori] church I have seen different people [come] who are HIV positive looking for treatment, looking for a cure. (Mark, man aged 30, HIV positive)

According to the discussions much of the perceived therapeutic potential of the churches, especially in limiting re-infection, lies in the moral policing of their adherents (Gregson *et al*. 1999). Joy's comment reveals the pressure placed on people to declare themselves HIV free so as not to be considered beyond redemption or immune to prayer. This focus on moral and communal values as underpinning HIV prevention and cure reinforces the conviction that spiritual beliefs provide the only lasting solution to HIV. This idea has implications for perceptions about healing:

 … there are certain pastors who are discouraging people taking their ARVs [antiretrovirals] and saying that you should believe in prayer and God in order to survive so it depends on the individual. If you think maybe the prayer will set you free then you will do that … the white garment [Vapostori] churches discourage even people from attending hospitals. (Hope, woman aged 28, HIV positive)

From a biomedical and public health standpoint (and indeed, from the perspectives of a significant number of those interviewed), the spiritualisation of disease can allow the virus to continue to destroy the individual's immune system, facilitate its spread and cause other forms of harm:

 … when I was young I developed goitre in the neck and n'anga cut the goitre with the razor blade so imagine if they take the same razor blade used on me and use it on another person … as a way of releasing those evil spirits. (Bea, woman aged 35, HIV positive)

At first people thought that this disease was because of witchcraft … [Some people] they go to the traditional healers [and] … will be told ‘Go and sleep with small girls’ so that the disease will go away. (Lucy, woman aged 36, HIV positive)

…*kukaranaka* is an inheritance issue whereby after the death of a brother the other brother will take on his wife. (Byron, man aged 25, HIV status not declared)

I know many people who have been going to see traditional healers … because they were thinking that it [HIV] was the *runyoka* problem a man develops when he sleeps with someone else's wife. They think that if they go to a traditional healer they would be given a traditional medicine that will clean their stomachs by making maybe having a runny tummy. (Carol, woman aged 40, HIV positive)

Ritual cutting, the virgin rape myth (i.e. sex with a child to ‘cure’ AIDS), and wife inheritance were reported by the participants as being outmoded and dangerous. In the case of rape or child sex, they are also illegal. Several references were made to the goblin-type creature, *runyoka*, which is activated when men violate sexual norms. In effect a guardian of female sexuality, *runyoka* causes AIDS-like symptoms which can only be brought under control by traditional medicine (Taylor 2005:111). As the participants recounted, a person who is HIV positive thus faces various and contradictory messages about HIV. He or she may be portrayed as a sinner, promiscuous, disrespectful of tradition, unlucky or sexually deviant, and as cultural reference points these characterisations complicate treatment choices. For example, Mark's Vapostori church tells him that the best response to his HIV infection is to ‘pray [for] eternal life’, a spiritual conception which is reflected in the way in which he sees the virus in his body:

 … sometimes I feel as if something is moving within my blood… I don't know what the thing might be. I might feel the movement from my backbone but I don't see sometimes it. I might feel it from my hand and I will maybe look at my hand that time but I don't see anything. (Mark, man aged 30, HIV positive)

Mark's feelings illustrate how mixed messages and contradictory ideologies can create complications in understandings about how HIV acts within the body and influence how it is socially perceived and therapeutically treated. How such perceptions interplay with biomedicine and the clinical delivery of care are our next consideration.

### Techno-scientific conceptions of disease and care

Zimbabwe's massive ART roll-out has occurred with the lowest level of per capita HIV funding in sub-Saharan Africa (UNAIDS 2012) during years of hyperinflation, political contestation, economic crisis and healthcare shortages (Apollo *et al*. 2010; O'Brien & Broom 2010). Despite these problems attitudes to ART among the participants were overwhelmingly favourable and over half of them were enrolled, or planning to enrol, on ART. Their stories of health recovery were often dramatic:

I weighed about 44 kg and my CD4 count was 29 … [After ART] my weight is about 77 kg and my CD4 count is 465 … I have had TB as well and warts, herpes, shingles. Before every disease would affect you … the nerves would flame up and you had flu and cough every month and even my skin has recovered I used to have thrush on my hands legs … now I have recovered. (Maria, woman aged 42, HIV positive)

I was bed ridden for six months. I used to urinate in my blankets and I could not walk. I couldn't wake up by myself. After taking ARVs I recovered so by 2010, in two years, I was fully better. (Havison, man aged 47, HIV positive)

This achievement has not been without problems as the government has struggled with the funding and supply of ART drugs and related technologies to the hundreds of clinics that provide ART around Zimbabwe (Fraser, Ruark, Gorgens, James, Milanzi, Colvin, *et al.* 2010). For the 200,000 or more people needing to enrol in treatment, the resolution of these issues was a matter of life and death:

For three solid months I kept on going to the hospital but was told that there were no ARVs. They said they were concentrating on people who were on treatment already … [You] just have to persist and persist in going to the hospital otherwise you will die. (David, man aged 51, HIV positive)

[the] local clinic … had closed the files, but there was another sister in charge who saw us and saw the condition of my child, he was seriously ill. His CD4 was below 200 … that is how we were enrolled. (Carol, woman aged 40, HIV positive)

The contradiction of treatment for some where others are denied means that the recovering/living are doing so alongside others who are dying. One of the central themes within the interviews was the injustice that the participants considered inherent in the delivery of clinical-based biomedical treatment and the variations in care across populations and groups. The interviewees reflected on how subjective criteria such as compliance, ‘deservedness’, perceived need, treatability, financial ability, catchment area and persistence determined access to care. These criteria also separated patients into impersonal medical categories such as tested/untested, treated/untreated, healthy/diseased and high/low CD4 count. Significant concerns were also evident in the participants' reports about the overall quality and availability of health services. One in three of those interviewed mentioned side effects such as swollen legs, vomiting, weight gain, ‘getting fat’, enlarged glands, constipation, diarrhoea, skin diseases, mobility, headaches, dizziness, inflamed ball joints, blindness, deafness, stroke and ‘brain problems’. All of these generated a certain apprehension about enrolment:

 … I don't want to consume pills each and every day and also I fear the issue of reaction [or] the side effects … if you do have some other diseases like diabetes … [and] you start consuming ARVs … those diseases would come out at the end those people would even not be able to walk. (Ethel, woman aged 38, HIV positive)

Aside from several who had been able to negotiate better healthcare access through NGOs, clinical trials or personal benefactors, very few of the participants were undergoing regular health monitoring. This was evident in their worries about the strains being placed on their general health:

 … my CD4 count was last checked two years ago … yet when we started they said you had to have your CD4 count checked after every three month. (Hazel, woman aged 36, HIV positive)

ARVs only suppress the virus they don't cater for the OI [opportunistic infections] so you need the clinic you need the primary doctor … we have a shortage of doctors. Mostly the OI clinics are staffed by student doctors. (Filomena, woman aged 34, HIV positive)

 … earlier this year the death rates increased for people living with HIV … because there was none of those tablets … If you are HIV positive you don't want to be stressed that way time after time … yesterday there were no drugs. (Stan, male, aged 50, HIV positive)

What I have noticed of people who take ARVs is that they need much food so one might be enrolled on ARVs but they might also fail to get enough food so that person is not guaranteed to a long life. (Noah, man aged 29, status not declared)

While ART has hugely shaped the profile and impacts of HIV in Zimbabwe, it is well documented that pharmaceutical interventions have only limited effects if not effectively monitored and partnered with a reasonable standard of living (Castro & Farmer 2005; Farmer, Nizeye, Stulac & Keshavjee 2006; Mhlanga-Gunda 2010). As the aforementioned participants, and others who were interviewed explained, local socio-economic constraint means that patient health monitoring (regular blood counts, professional medical consultations, guaranteed drugs supply, etc.) may have been severely compromised and limited. Monitoring also depends on the availability of trained staff to oversee clinical services (Hallett *et al*. 2011). As Noah observed diet is also important for ARV intake, not only to assist with drug ingestion but also for the overall nutrition and well-being of the patient (Gwatirisa & Manderson 2009:108). However, one-third of the participants stated that they could not afford an adequate or balanced diet and, moreover, found it difficult to meet the costs associated with treatment. It was clear that such structural factors can shape biomedical treatment to be seen as just another competing form of HIV treatment and management. The consequences can be seen here:

I don't want to know my status because I am living in poverty and by the time that I get to know that I am HIV positive I am not in a position to buy the pills … or going through those [enrolment] processes as they might require money so I [don't want] … to know my status. (Noah, man aged 29, status not declared)

The ARVs didn't work for my children … they were all enrolled on ARVs … but it just didn't work out for them so in the end I just concluded that they were destined [to die early] … (Portia, woman aged 63, HIV positive)

In the previous 10 years, Portia had lost all of her seven children. Their death certificates recorded asthma, TB, pneumonia, meningitis, gastroenteritis and ‘immune deficiency’, yet she attributes their deaths not to poverty and treatable disease but to fate: ‘I just concluded that they were destined’. Despite encouragement from his wife, Noah was worried that being tested or commencing ART could bring on fatal illness. He assumed he was positive as he had shared a prison cell with 60 others during a two-year sentence and many of his friends and neighbours, including his brother, had died of AIDS. Noah also reflects another form of cultural gendering acknowledged by participants of both sexes in that men, discouraged by representations of masculinity (especially social pride), are inclined to avoid HIV testing and treatment (Campbell, Skovdal, *et al*. 2011; Skovdal, Campbell, Madanhire, *et al*. 2011).

Also present throughout the interviews was reference to worries such as rental payments, earning a living, school fees, finding and keeping lodgings and coping with stigma. Such pressures were reported as being best to avoid: for example, ‘without stress I will live longer’ – Havison, ‘people … die out of stress’ – Sara and ‘HIV doesn't want stress’ – Stan. Treatment anxieties, the CD4 test, ARV shortages, waiting times, fees and charges and transport fares added further dimensions to these disquiets and speak to a social marginality which a purely techno-scientific biomedical model cannot resolve. The systematic denial of access to the essentials of life is a form of structural violence (Farmer *et al*. 2006) which causes social and individual harm. Originating out of economic, political and historic processes, this negation renders the poor more vulnerable to both infection and illness (Castro & Farmer 2005) and adds an additional layer of significance to the dialectic between the biomedical clinic and traditional medicine and supernatural forms of healing, which we shall now discuss.

### Traditional medicine and biomedical treatment: contestations and collaborations

The interview data confirmed existing reports (Nguyen *et al*. 2011) that even under stable social conditions the deployment and functioning of biomedical health technologies can present significant resourcing problems (GoZ 2012). In circumstances such as those outlined by the participants, the patient can experience biomedicine as mystifying, time wasting and bureaucratic. ART enrolment procedures, for example, involved a series of lengthy tests which needed to be completed before being prescribed the ‘starter pack’, the initial drug combination that Wilbert described as being intended to ‘see if you die or get better’. Given these framings, and the limitations in the reach and delivery of ART, the participants indicated their openness to various treatment options. The popularity and visibility of traditional medicine in Zimbabwe, for example, is evident in the sale of alternative remedies in pharmacies and super markets. In addition, as a consequence of having had a long history of cultural engagement and adaptation, traditional medicines and healing systems were reported as coexisting with biomedicine. According to the literature, herbal and traditional remedies are strongly supported by people living with HIV (Bepe, Madanhi, Mudzviti, Gavi, Maponga & Morse 2011; Pearson & Makadzange 2008; Taylor 2005) despite the fact that their biological effectiveness is unproven (Bepe *et al*. 2011; Taylor 2005). On an official level legislation and regulation have institutionalised traditional medicine (MoHCW 2007) as evident in the discourse of Clarence, a traditional medical practitioner:

 … someone who is HIV positive he comes at my surgery he says … ‘*Sekuro* doctor I am suffering from diarrhoea’ we can treat that diarrhoea we can stop it with herbs. Someone can come ‘I am coughing’ we give him African herbs to cure that cough but as a traditional medical practitioner I can't treat AIDS because AIDS … is caused by a virus which kills the white cells … we can control but not kill the virus. (Clarence, man aged 54, status not declared)

Herbal remedies, which aim to boost the immune system and alleviate symptoms, provide an entry point for patients who might otherwise be sceptical of traditional medicine. In addition, participants observed that the community-based and locally resourced healer could offer more accessible and private treatment than the often distant, under-resourced and expensive biomedical clinic (Pearson & Makadzange 2008). Taylor (2005), for example, found that traditional healers provide the majority of care and treatment for HIV patients. However, at least in Harare in 2010, they were not always highly regarded:

 … people sometimes skip the ARVs by going to a witch doctor, the traditional healers but they are only wasting their time, even [immune] boosters … I was given the recipe for those herbs the Moringa tree … It didn't work quite well with me. (Wilbert, man aged 45, HIV positive)

 … what the traditional healer told me [about why I was not getting a promotion] was a load of rubbish. I just wanted to find out what happens in that part of the world. For HIV I went to a medical doctor as I knew it was beyond the scope of traditional healers. (David, man aged 51, HIV positive)

 … there is no traditional healer who can cure this disease. The only place where you can get a cure for this disease is the graveyard [laughs] … a lot of traditional healers they don't come out openly and say that they don't have a cure. Sometimes they will … refer you to the local clinic. (Gertrude, woman aged 69, HIV positive)

The whole family went [to the n'anga]. It was 15 years ago. It was for cleansing from the extended family but it was my immediate family only. We had two lost from my family, two from HIV and one was epileptic, my sisters took us. (Tawanda, woman aged 42, HIV positive)

While some of these accounts are referencing events which predate ART in Zimbabwe, they also indicate willingness, but not an uncritical one, to alternate between, or combine, different medical systems. The non-exclusive nature of traditional medicine in Shona culture is illustrated by how Clarence framed his discourse (‘I can't treat AIDS’) and demonstrated a clear disposition for partnership with biomedicine. This duality between traditional medicine and the clinical biomedicine is explored in the following comments:

…a friend of mine … married a pastor she was in our [AIDS] support group but … she believed that she has been healed so she stopped taking the ARVs but after some time she fell ill she died. (Juliet, woman aged 46, HIV positive)

 … I know a guy who was on ARVs and he went to a prophet and he was told that his own mother was the one who was causing the sickness and he stopped taking ARVs … He started to be very seriously ill … I think he is taking his ARVs now. (Helen, woman aged 43, HIV positive)

 … some people I know who have thrown away their ARVs but they now have side effects some have difficult skin rash and some they have to go again through the whole process of getting the ARVs again. (Bea, woman aged 35, HIV positive)

I stopped consuming these ARVs for about two years but then I started having problems with my body, I was having migraine headaches and stomach problems then I went to the clinic… and I was enrolled again as I am seeing that ARVs are effective … (Delia, woman, aged 41, HIV positive)

we were giving [my husband before he died] a lot of herbs mixed with the ARVs. (Filomena, woman aged 34, HIV positive)

 … this herb Moringa which a lot of HIV positive take … I was discouraged from taking the Moringa because it reduces the effectiveness of the [ARV] drug I consume. (Abigail, woman aged 41, HIV positive)

…people also they are attending both the hospital … and the traditional medicine and the hospital medicine again. (Norman, man aged 33, HIV positive)

This pattern of ‘in and out’ of the clinic can be observed in the adherence rate whereby 15% of new ART patients stopped taking their drugs within 12 months (GoZ 2012). Participants who spoke about treatment interruption generally did so in the context of allegiance to alternative therapies, particularly spiritual healing. Delia was an exception, her two-year break from ART was provoked by a disagreement with clinic staff about counselling. Eventually the nurses persuaded her to resume ART. Unfortunately for Julia's friend, the pastor's wife, her continued adherence to spiritual healing led to her death. The promise of a cure and the stigma of being known to take ART (especially for men) added to the overall appeal of traditional medicine and spiritual healing. Some respondents, especially those with a strong Christian and spiritual healing orientation, tended to demean traditional medicine, as seen with Byron's comment that ‘with the rise of Christianity… traditional healers are now being regarded as evil’. In addition, the spiritual healing churches were reported to be intolerant of other treatment options. Juliet, for example, who attends a Pentecostal ministry, comments ‘the Holy Spirit can kill the virus … if you are prayerful you can be healed’. While the participants expressed different opinions as to the role of various churches in helping prevent HIV and treat AIDS, there was general support for Isaac's remark that ‘Spiritual counselling is quite nourishing’.

An element of the dynamic relation between biomedicine and alternative healing systems (both Christian and traditional) is illustrated by the way in which the latter can either support existing care (Campbell, Scott, *et al*. 2011) or channel a patient into (and out of) the clinic (Skovdal, Campbell, Nhongo, *et al*. 2011). It was also apparent from the participants' stories that traditional medicine, as diverse as this category is, has incorporated biomedicine into its practice (Pearson & Makadzange 2008). As these particular beliefs position illness (and cure) as emergent from the spiritual, they may also condone what public health and biomedical practitioners (and many of the participants) consider to be dangerous practices. In addition, as we have outlined earlier in the paper, they may also promote approaches to healing which complement rather than contend with ART. In these contexts, non-biomedical choices may be valuable and culturally important practices to particular people. While an ‘ART is best’ model (Apollo *et al*. 2010) may work well from an epidemiological position, the dismissal or lack of acknowledgement of traditional beliefs and practices and forms of localised superstition can evade the realities of significant limits to biomedical care and the potencies of grassroots belief and tradition. Furthermore, the treatment-as-prevention focus, based on the idea that ART reduces viral load and infectivity (Cohen, Chen, McCauley, Gamble, Hosseinipour, Kumarasamy, *et al.* 2011), de-emphasises community-based attitudes to, and practices of, HIV prevention (Nguyen *et al*. 2011). For example, it was evident from the participants' accounts that community conversation about HIV, motivated by illness and funerals, helped people realise the need to avoid infection, and this has contributed to the easing of the HIV epidemic in Zimbabwe in recent years (Muchini, Benedikt, Gregson, Gomo, Mate, Mugurungi, *et al.* 2011). In addition, as also told by the participants, the dramatic impact of ART patients being seen to be literally rising from their death beds lead people, including religious leaders, to overcome reservations, spiritual or otherwise, and either openly or covertly opt for biomedical options.

## Discussion

The roll-out of ART underway in economically poorer countries is clearly a central public health priority. However, it is also important to recognise that these technologies are being inserted into a context of a significant diversity of belief and traditional practices. While there has also been increasing attention paid in the literature to the interplay between traditional medicine, biomedicine and HIV in developing countries, this research has tended to focus on collaborative opportunities between different health systems and the possibility of adverse reactions to ART (Babb, Pemba, Seatlanyane, Charalambous, Churchyard & Grant 2007; Littlewood & Vanable 2011; Mudzviti, Maponga, Khoza, Ma & Morse 2012; Shuster, Sterk, Frew & del Rio 2009; Tamuno 2011; Wreford 2005). These studies also show that patients with serious health problems in developing countries face varied structural, practical and cultural challenges that may result in service provision gaps being filled (albeit problematically at times) by traditional medicine and spiritual healers. They also indicate that the pharmacological potency of antiretroviral drugs can promote among clinicians a lack of consideration of the role of other care practices. This is despite the fact that structural problems (e.g. limits to ART coverage) and cultural ideas about techno-scientific medicine (e.g. the historic presence of pluralistic health practices) may limit the practical effectiveness of ARV drugs. The lived experience of HIV can tell us various (important) things about attitudes to illness, healing and traditional/spiritual health practices. The perseverance of superstitious and existential solutions, for example, may be embedded in persistent misconceptions of transmission (e.g. personal responsibility and moral breakdown) and lack of structural support (e.g. economic deprivation, infrastructure limitations and technical shortages). Certainly the durability of traditional practices cannot be written off as merely ‘stage in development’ phenomena. In Zimbabwe spirit mediums and diviners, as well as traditional medicine, are important elements of nationalist iconography forged during the liberation struggle (Lan 1985) and illustrate the place of tradition within the political economy of the State. Biomedical treatment is thus situated within a cultural context of enduring religious influences, structural and political problems and histories of care and value. Moreover, ART has been presented in a setting whereby biomedicine is viewed as both fractured and delivered with less efficiency and deference than we see in some western contexts. While the participants reported that they adapt and utilise whatever forms of healing are available and affordable, their preferences are based on ‘what works best’. Ultimately, both forms of health practices have a place (albeit some rather precariously) and thus form part of the therapeutic landscape of contemporary HIV care in Zimbabwe. Understanding the complex intermingling of biomedicine (primarily ART and clinical surveillance) with traditional medicine and spiritual practices, based on the lived experience of recovery, can allow us to move beyond a largely epidemiological approach to managing the epidemic. The referral practices which are already employed by some healers, for example, could provide a platform for collaboration and hence communication between healers and medical practitioners. This possibility, indeed the whole question of treatment pluralism, is missing from the national AIDS strategy (National AIDS Council 2011) and indicates a lack of appreciation that barriers to ART implementation can lie very much in the forms of patient engagement. Traditional beliefs and practices do not necessarily wane when seemingly effective biomedical interventions are introduced to the general population. Consequently, patients and their carers need to be made more aware of the potential risks, and benefits, of complementary and alternative healing. Policies which encourage dialogue between healers and clinicians, and awareness among health workers, can help to ensure that more informed decisions around drug efficacy and incompatibility (what works and what does not) are made. Conversations about therapeutic choices ultimately need to occur among patients and their carers so that messages are conveyed effectively. HIV as a condition and AIDS as a disease should thus be viewed within a context of politics, belief, physiology and socio-economic deprivation. While witchcraft, spiritual healing and religious superstition may appear to be niche issues within a wider economic of care, they still shape and influence peoples’ lived experiences of HIV. In this sense, they must be central to any contemporary understanding of the problem of how to make life-saving therapies efficiently available on a mass scale in a way that is not just tied to drugs but holistically to economic empowerment, food security, nutrition and overall well-being. The contribution of this study has been to advocate engagement with traditional and spiritual healing in a way that recognises that they are aspects of culture, individuality and community identity which add meaning to people's lives, and should not be simply regarded as barriers to compliance.

## Note

1. In accordance with UNAIDS (2011), this paper employs the terms AIDS and HIV separately according to the specific context.
